# The value of real world evidence and pragmatic trials in advanced prostate cancer- insights from the electronic Prostate Cancer Australian and Asian Database

**DOI:** 10.3389/fonc.2024.1494073

**Published:** 2024-12-09

**Authors:** Angelyn Anton, Kristina Zlatic, Sophie O’Haire, Ben Tran

**Affiliations:** ^1^ Personalised Oncology Division, Walter and Eliza Hall Institute of Medical Research, Melbourne, VIC, Australia; ^2^ Cancer Services Department, Eastern Health, Melbourne, VIC, Australia; ^3^ Department of Medical Oncology, Peter MacCallum Cancer Centre, Melbourne, VIC, Australia

**Keywords:** prostate cancer, real world data, registry trials, pragmatic trials, older patients

## Abstract

Prostate cancer is a common malignancy with an increasing incidence in ageing populations. However, older patients with prostate cancer are often underrepresented in traditional clinical trials. The electronic Prostate Cancer Australian and Asian Database (ePAD) is a multi-centre, multi-national prospective clinical registry, that records real world data from a broader population. An analysis of the first 753 metastatic castration-resistant prostate cancer (mCRPC) patients within ePAD demonstrated that 43% were aged 75 years and older. Older patients were more likely to have comorbidities including ischemic heart disease, diabetes and previous stroke. Treatment outcomes were similar in all age groups. However, older patients receiving chemotherapy were more likely to stop treatment due to toxicity. Furthermore, in a smaller ePAD analysis involving additional chart reviews within 3 high volume centres, at least one relative or absolute contraindication to abiraterone was seen in 72% of our cohort and with enzalutamide in 14%. In total, 47% had potential clinically significant drug interactions with abiraterone and 67% with enzalutamide. Registry-based randomised controlled trials (RRCTs) are a novel trial methodology aiming to bridge the gap between retrospective registry analyses and traditional randomised controlled trials. We conducted the REAL-Pro study in advanced prostate cancer, comparing cognition, depression and falls risk between CRPC patients receiving abiraterone or enzalutamide. The study closed early due to slow recruitment and a changing treatment landscape, highlighting the need for further research to understand clinician and patient perspectives towards pragmatic trials such as RRCTs and subsequently develop strategies to optimise future trial design and recruitment.

## Introduction

Prostate cancer is the most common non-cutaneous cancer among men in developed countries with an incidence that increases with age (1, 2). The ageing population is increasingly placing greater burden on health systems and the expected increase in prostate cancer incidence is a significant global issue. While the treatment landscape in advanced prostate cancer has rapidly evolved, a major clinical challenge is the management of older adults with competing comorbidities and complex psychosocial care needs. An analysis of the SEER database demonstrated that although prostate cancer outcomes have improved in recent years overall, outcomes remain unchanged in the subgroup of those over 75 years (3). In the real world setting, clinical decisions are made by extrapolating from randomised controlled trial data. However, these are often limited by narrow eligibility criteria, which impacts the generalisability of results. A Food and Drug Administration survey highlighted the underrepresentation of older adults over the age of 75 years, who represented only 12% of clinical trial participants between 2005 and 2015 despite comprising almost 30% of new cancer diagnoses (4).

Real world databases allow comprehensive data collection on a large scale to allow prospective data collection and analysis of a broader, representative population. These data enable better understanding of real-world patient and disease characteristics, treatment selection patterns, efficacy and safety outcomes in comparison to those of clinical trial populations. Previous studies have suggested an effectiveness-efficacy gap due to these differences between populations, whereby progression-free and overall survival estimates in real world studies have been inferior to those of the original trial populations (5). However, more contemporary datasets have demonstrated equivalent outcomes, likely relating to the advances in supportive care, subsequent therapies becoming available and accessible as well as lead time bias, with more accurate diagnostic modalities or earlier detection through more frequent surveillance leading to apparent survival improvements (6). Real world databases may also be leveraged for a range of purposes, including linkage with external datasets or the conduct of prospective clinical trials (7).

## Real world data in advanced prostate cancer

The electronic Prostate Cancer Australian and Asian Database (ePAD) is a multi-centre, multi-national prospective clinical registry, designed to collect pertinent information relating to consecutive real world patients with advanced prostate cancer. The database initially commenced as an investigator-initiated study in 2016 and had been designed to focus on castration-resistant prostate cancer (CRPC), given the treatment landscape at that time. Subsequent updates have enabled collection of data relating to treatment intensification in the metastatic hormone-sensitive prostate cancer (mHSPC) setting as well as detailed information regarding molecular testing and the presence of DNA-repair defects, following the emerging evidence supporting life-prolonging therapies in both settings. Currently, ePAD collects data from 23 sites, including 5 within regional or rural locations and 8 private practice settings. Most recently, 3 international sites within Asia have also been included with ongoing plans to involve additional sites.

Data are entered by clinicians or trained data abstractors into a password-protected online database and updated at regular intervals. All patients treated at the participating treatment site(s) are eligible and a waiver of consent was granted by the Melbourne Health human research and ethics committee, given the collection of standard information that is available in pre-existing medical records. A steering committee, including the top 5 recruiters for the previous calendar year, reviews and evaluates the scientific merit of any proposals for projects. All data are housed on a secure server and only combined data summaries are provided to investigators with all data published in non-identifiable form. To date, 1935 patients have been enrolled and there have been 19 publications from registry-based projects that have demonstrated informative findings and address important clinical questions.

## Treatment patterns and outcomes in older men with prostate cancer

Given the under-representation of older patients with prostate cancer in conventional clinical trials, this population has been an area of interest within several ePAD registry-based projects. One such project compared treatment choices and outcomes between the <75 year age group and those aged 75-85 years and >85 years (8). Within ePAD at the time of analysis, there were 753 patients with mCRPC, of whom 327 (43%) were aged 75 years and older at the time of castration-resistance, including 90 (12%) who were over 85 years. Older men aged 75 years or greater were more likely to have comorbidities including ischemic heart disease, diabetes and previous stroke than the younger group (**Table 1**). Treatment patterns were also different, with younger patients more likely to receive more than one line of systemic therapy for metastatic CRPC (mCRPC) as well as prior docetaxel for mHSPC and older patients were more likely to receive androgen receptor pathway inhibitors (ARPI). While treatment outcomes including time to treatment failure, PSA response rates and overall survival were similar for all treatment groups with mCRPC, older patients who received chemotherapy were more likely to stop treatment due to toxicity (8).

**Table 1:** Baseline characteristics by age group.

	<75y (n=426)	75-85y (n=237)	>85y (n=90)	P-value
Median Age	66.6y	79.6y	88.0y	
*Patient Comorbidities*				
Ischemic Heart Disease	68 (16%)	70 (30%)	37 (41%)	**<0.001**
Stroke	22 (5%)	25 (11%)	11 (12%)	**0.024**
Peripheral Vascular Disease	9 (2%)	13 (6%)	7 (78%)	**0.020**
Hypertension	211 (50%)	142 (60%)	66 (73%)	**0.002**
Hypercholesterolaemia	144 (34%)	104 (44%)	46 (51%)	**0.036**
Diabetes	57 (13%)	58 (25%)	13 (14%)	**0.003**
Cognitive Impairment	12 (3%)	7 (3%)	6 (7%)	0.182
Smoking History	157 (37%)	68 (29%)	36 (40%)	0.194
*Disease Characteristics*				
Gleason Score				
< 8	95 (22%)	68 (29%)	16 (18%)	**0.003**
≥ 8	231 (54%)	86 (36%)	21 (23%)	
Unknown	100 (23%)	83 (35%)	53 (59%)	
De Novo Metastatic Disease	112 (26%)	42 (18%)	18 (20%)	**0.033**
Visceral Metastases	36 (9%)	21 (9%)	7 (8%)	0.951
Median PSA at mCRPC diagnosis	54.25	64.44	81.18	**<0.001**
PSA Doubling Time at mCRPC diagnosis				
≤3months	224 (53%)	113 (48%)	30 (33%)	**0.014**
>3months	114 (27%)	76 (32%)	33 (37%)	
Unknown	88 (21%)	48 (20%)	27 (30%)	
Prior Curative-Intent Treatment for Localised Disease	247/314 (79%)	118/195 (61%)	31/72 (43%)	**<0.001**
Prior Upfront Systemic therapy for mHSPC				
Docetaxel	87 (20%)	16 (7%)	0	
Abiraterone	1 (<1%)	2 (1%)	0	
Enzalutamide	4 (1%)	3 (1%)	0	

p values that are statistically significant (<0.05) are displayed in bold.

Drug-drug interactions and comorbidities are also important considerations among patients with mCRPC that influence treatment selection in the real world. Through comprehensive chart reviews of patients included in ePAD from 3 high volume sites, we evaluated the impact of concomitant medications and comorbidities on 235 patients with mCRPC (9). Our data demonstrated that 83 (72%) of 116 patients on abiraterone had at least one relative or absolute contraindication relating to a documented baseline comorbidity as did 17 (14%) of 119 patients receiving enzalutamide (**Table 2**) (9). Both abiraterone and enzalutamide have the potential to interact with other drugs due to the influence of cytochrome p450 enzymes. Within our cohort, 55 (47%) had potential clinically significant interactions with abiraterone and 90 (67%) with enzalutamide. Potential drug interactions included common classes such as antidepressants and anticonvulsives with abiraterone as well as novel anticoagulants and antiarrhythmic agents with enzalutamide. Importantly, those who were receiving enzalutamide together with potentially interacting drugs, had an inferior overall survival compared to those without interactions (9), highlighting the important clinical implications of our findings. These data are particularly relevant to the use of ARPI in older patients, who are more likely to have pre-existing comorbidities and to experience polypharmacy.

**Table 2:** Baseline characteristics of patients treated with first- or second-line abiraterone acetate (AAP) or enzalutamide (ENZ) in mCRPC.

Characteristics	1st line		2nd line	
	AAP n = 87	ENZ n= 88	AAP n = 29	ENZ n = 47
Median age at ARPI initiation, years	78	73	74	73
ECOG, n (%)				
0	49 (56)	67 (76)	11 (38)	34 (72)
1	29 (33)	14 (16)	13 (45)	11 (23)
> 2	8 (9)	5 (6)	4 (14)	2 (4)
Gleason Score, n (%)				
7	13 (15)	16 (18)	7 (24)	9 (19)
8	9 (10)	6 (7)	6 (21)	8 (17)
≥9	32 (37)	34 (39)	10 (34)	19 (40)
Co-morbidities, n (%)				
Hypertension	52 (60)	52 (59)	15 (52)	30 (64)
Hypercholesterolaemia	34 (39)	37 (42)	14 (48)	22 (47)
GORD or peptic ulcer disease	22 (25)	18 (20)	6 (21)	11 (23)
IHD	18 (21)	17 (19)	8 (28)	8 (17)
Diabetes	13 (15)	18 (20)	8 (28)	8 (17)
Atrial fibrillation and other arrhythmia	21(24)	13 (15)	3 (10)	7 (15)
Depression or anxiety	13 (15)	9 (10)	5 (17)	6 (13)
Cognitive impairment	8 (9)	1 (1)	2 (7)	1 (2)
Stroke	7 (8)	4 (5)	3(10)	2(4)
Heart failure	7 (8)	8 (9)	2 (7)	4 (9)
Falls	4 (5)	1 (1)	0 (0)	1 (2)
Parkinson’s disease	4 (5)	0 (0)	0 (0)	1 (2)
Traumatic brain injury	3 (3)	0 (0)	1 (3)	1 (2)
Seizures	2 (2)	0 (0)	0 (0)	0 (0)
CCI, median	10	9	10	9
Conmeds, median (range)	4 (0-19)	4 (0-18)	6 (0-15)	5 (0-15)

Comorbidities with potential to interact with respective androgen receptor pathway inhibitors (ARPI) are shaded in orange.

CCI, Charlson co-morbidity index; conmeds, concomitant medications; ECOG, Eastern Cooperative Oncology Group performance status; GORD, gastro-oesophageal reflux disease; IHD, ischaemic heart disease.

## Registry-based randomised controlled trials

As demonstrated through previous ePAD analyses, registry data can address clinically relevant questions and provide insights to guide treatment decision making in evidence-free scenarios. However, these data are often limited by their retrospective nature and the risk of selection bias or other confounding factors that are difficult to adjust for statistically. Therefore, registry-based randomised controlled trials (RRCTs) are a novel trial methodology aiming to bridge the gap between retrospective registry analyses and traditional randomised controlled trials (10). RRCTs have the statistical robustness of a traditional randomised study, including the ability to stratify based on important prognostic factors but allow more pragmatic trial designs and generally include a broader real-world population to increase external validity and generalisability of the results. Given the use of an established database, including pre-existing logistics, governance and infrastructure for prospective data collection, RRCTs can also be conducted at a much lower cost (10).

## REAL-Pro: a registry-based randomised study of enzalutamide vs abiraterone assessing cognitive function in elderly patients with metastatic castration-resistant prostate cancer

Given the clinical equipoise relating to the optimal ARPI treatment choice in older men with prostate cancer, we developed the first RRCT in advanced prostate cancer to address this question.

**Figure 1:**
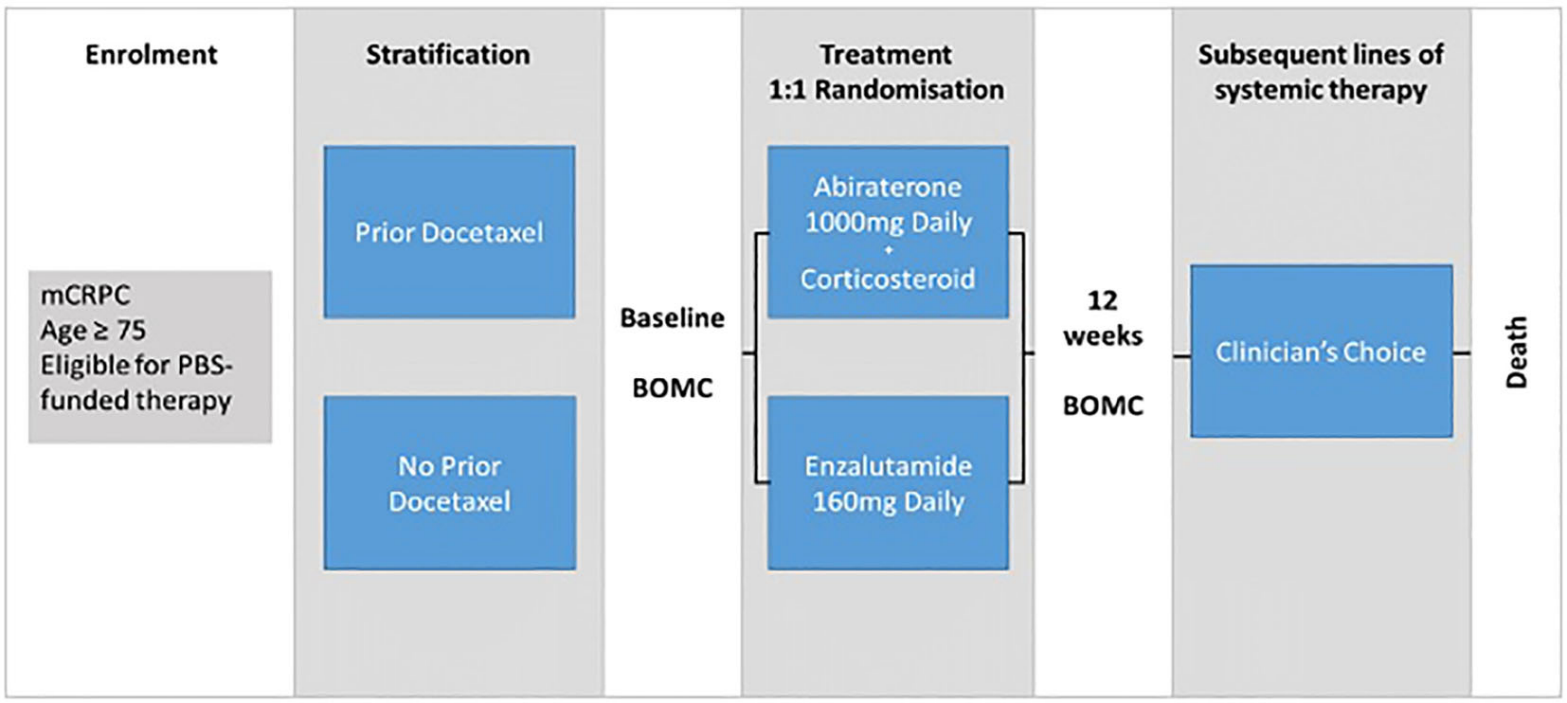
REAL-Pro study schema. mCPRC, metastatic castration-resistant prostate cancer; BOMC, Blessed Orientation-Memory-Cognition cognitive assessment tool; PBS, Pharmaceutical Benefits Scheme; Clinician's choice, Treatment decisions regarding subsequent therapy made by clinician at local treating site, no protocol-based restrictions, decisions based on local standard of care protocols.

REAL-Pro is a RRCT utilising the existing ePAD clinical registry for prospective data collection. Patients aged 75 years or older who were suitable to receive either abiraterone or enzalutamide via the Australian Pharmaceutical Benefits Scheme (PBS) for mCRPC were eligible for inclusion into the study. The PBS subsidises monotherapy in ARPI-naïve patients with mCRPC before or after docetaxel chemotherapy. Patients were randomized to either ARPI and stratified based on prior docetaxel chemotherapy use, given the potential longer-term effect of chemotherapy on cognitive function (**Figure 1**). Real-time randomisation was performed by a central study co-ordinator to facilitate timely commencement of systemic therapy.

Prior studies had suggested that enzalutamide is associated with an increased risk of neurocognitive side effects including a greater change from baseline cognition within the first 3 months of therapy- with subjective and objective tools (11, 12). However, this study included patients of all ages and subsequently, Khalaf et al. demonstrated that men aged 75 years or older had inferior quality of life compared to those on abiraterone in that age group, whereas there were no differences in those under 75 years (13). Based on the existing literature, REAL-Pro therefore aimed to evaluate changes in cognitive function within 3 months of therapy in those aged 75 years or older.

A phone interview was conducted at baseline and after 12 weeks of treatment by the central study co-ordinator using validated tools including the Blessed Orientation Memory Cognition tool to assess for cognitive impairment, the Geriatric Depression Scale, a self-reported screening tool for depression and the Falls Risk Questionnaire assessing the risk and incidence of recent falls. The pragmatic design comparing two standard of care therapies required minimal additional resources from study sites and the frequency of clinical reviews, pathology and imaging investigations were left to clinician discretion. Following progression, any subsequent therapies were permitted based on the local treating clinician’s choice, with these data being prospectively collected and updated according to the existing ePAD registry protocol.

We enrolled 76 men between June 2019 and September 2023. However, recruitment ceased in December 2023 due to slow accrual and the changing treatment landscape with the increasing use and availability of ARPI in the earlier mHSPC setting. While 56 (74%) patients completed both telephone assessments, statistical analyses were limited by this small sample size. REAL-Pro therefore demonstrated potential barriers to the effective conduct of RRCTs. It is hypothesised that in the real world setting, many patients have greater suitability for one ARPI over another, reducing the pool of eligible patients for randomisation in this study. This is consistent with our retrospective analyses demonstrating a high incidence of potential comorbidities and drug interactions within the real-world population of patients receiving ARPI. An important benefit of conducting this trial within an existing registry is that further analyses can be performed to include all patients within our target study population who have been enrolled within ePAD and commenced ARPI treatment during the recruitment period. These data will help to determine potential clinical factors influencing recruitment and offer valuable real-world insights that may influence clinical practice.

## RRCT challenges and future directions

While having trials embedded within a registry enables the identification of patient-related factors that may have impacted recruitment, such as those relating to eligibility criteria or suitability for randomisation, factors relating to clinician perspectives are more challenging to ascertain. Previous qualitative research has suggested several self-reported challenges associated with effective RRCT implementation, including competing clinical trials, the perception of RRCT study questions being “less exciting” and the wish for greater renumeration despite the reduced resource burden (14). RRCTs predominantly address research questions relating to the use of standard of care therapies where there is sufficient equipoise, which are generally less common than research questions relating to novel interventions. However, trials with novel therapies are less feasible to conduct as RRCTs without the addition of numerous resources including study staff such as clinical trial pharmacists and more comprehensive data collection, including the reporting of adverse events, which may be more appropriate to conduct as traditional randomised controlled trials. However, the role of RRCTs in informing clinical practice and health policy is becoming increasingly recognised, with the potential to improve recruitment efficiency, data completeness with reduced cost and carbon footprint (15, 16). A further qualitative analysis is planned to develop greater understanding of clinician and patient attitudes towards RRCTs, through interviews of those involved in the REAL-Pro study and to identify potential barriers to successful conduct and strategies to improve recruitment to future trials.

## Conclusion

Real world data offer unique perspectives and have the potential to address clinically relevant research questions. Pragmatic trials are a novel trial methodology with potential to bridge the gap between the gold standard randomised controlled trials and real world evidence. The real world management challenge of older men with prostate cancer provides the ideal setting for the conduct of pragmatic trials. The REAL-Pro RRCT demonstrated the potential utility of clinical registries to answer important real world clinical questions. However, the slow recruitment also demonstrated the need to better understand barriers to implementation. Further research, particularly focusing on clinician-driven factors that influence recruitment, will enable development of strategies to engage investigators and optimise future pragmatic trial designs.

## Data Availability

The raw data supporting the conclusions of this article will be made available by the authors, without undue reservation, upon request with a data transfer agreement.
